# Horizon 2020: European perspectives in healthcare sciences and implementation

**DOI:** 10.1186/1878-5085-5-S1-A1

**Published:** 2014-02-11

**Authors:** Irene Norstedt

**Affiliations:** 1 Head of Unit “Personalised Medicine”, European Commission, Brussels, Belgium

## 

The potential benefits that preventive, predictive and personalised medicine approaches can bring to healthcare are discussed with much interest throughout the EU. This novel approach is based on a better understanding of the molecular mechanisms of health and disease and it has now begun to show results. There is, however, a long way to go before the area is fully exploited. Although the specific focus on personalised medicine is relatively new, the European Union has already committed significant funding to research in enabling technologies relevant to the field, as well as to specific disease areas for their application. More than € 1 billion has been committed to this research through the Seventh Framework Programme for Research and Technological Development (FP7, 2007-2013). A stocktaking exercise which started in 2010 has helped identify immediate and future challenges in personalised medicine research which will also inform the new EU research funding programme Horizon 2020 (2014-2020). The Commission's proposal for Horizon 2020 has a focus on societal challenges that should allow a higher degree of integration of different fields of research. This approach will cover activities from research to the market in an effort to bridge the current gap between the two. In this respect, the *Health*, *Demographic Change and Wellbeing Challenge* fits well the need for a multidisciplinary approach to the field of personalised medicine which is essential to move the area forward.

